# Pediatric-onset limited ANCA-associated vasculitis arising during pre-existing chronic recurrent multifocal osteomyelitis

**DOI:** 10.1186/s12969-023-00876-x

**Published:** 2023-08-24

**Authors:** Esraa Eloseily, Michael Henrickson

**Affiliations:** 1 Division of Pediatric Rheumatology, Cincinnati Children’s Hospital Medical Center, 3333 Burnet avenue, Cincinnati, OH 45229 USA; 2 Division of Pediatrics, Assiut University School of Medicine, Assiut, Egypt

**Keywords:** Granulomatosis polyangiitis, Chronic recurrent multifocal osteomyelitis, Pyoderma gangrenosum

## Abstract

**Background:**

Granulomatosis with polyangiitis (GPA) is an autoimmune disease characterized by chronic vasculitis involving small to medium sized arteries, granulomatous inflammation of the upper and lower respiratory tracts, pauci-immune necrotizing glomerulonephritis, as well as vasculitis of other organs. Chronic recurrent multifocal osteomyelitis (CRMO) is an autoinflammatory syndrome characterized by sterile bone inflammation.

**Case presentation:**

We report a case of CRMO that was doing well on non-steroidal anti-inflammatory drugs (NSAID for 6 years and then developed ANCA positive limited GPA presenting with pyoderma gangrenosum, persistent bilateral otalgia with serous otitis, otorrhea, then sensorineural hearing loss.

**Conclusion:**

This is the first report of limited GPA initially presenting as pyoderma gangrenosum in a patient with underlying CRMO. It is unclear how the pathology of an autoimmune and an autoinflammatory condition can overlap.

**Supplementary Information:**

The online version contains supplementary material available at 10.1186/s12969-023-00876-x.

## Background

Granulomatosis with polyangiitis (GPA), a chronic form of ANCA (anti-neutrophil cytoplasmic antibody) associated vasculitis (AAV) involving small to medium sized arteries, is primarily characterized by granulomatous inflammation of the upper and lower respiratory tracts and pauci-immune necrotizing glomerulonephritis, as well as the potential for other organ vasculitis. GPA is rare in children, with rising incidence in adults. In the pediatric population, disease onset usually arises during the second decade of life, affecting females more than males. Characteristic clinical presentation involves a triad of inflammation in the upper and lower respiratory tracts, along with renal disease [1]. Limited GPA is a milder form commonly isolated to the upper respiratory tract [2]. The diagnosis of GPA relies on a combination of clinical manifestations, the detection of specific serological markers (mainly ANCA, particularly PR3-ANCA or cANCA), and distinct histopathological findings (such as necrotizing granulomas of the upper and/or lower respiratory tract, inflammation in small to medium arteries, capillaries, or small veins, or pauci-immune glomerulonephritis) [3]. Chest imaging usually shows nodular and fixed infiltrates or cavitations [4]. Treatment of GPA depends on organ involvement and severity. More severe disease necessitates the use of stronger agents such as cyclophosphamide and high dose corticosteroids [5]. Remission and relapse rates, as well as adverse events were found to be similar rituximab and cyclophosphamide [6, 7]. In addition, rituximab was found to be efficient in re-inducing remission after relapse [8]. In addition, mycophenolate mofetil was found to be non-inferior to cyclophosphamide in inducing remission in patients with estimated GFR > 15 ml/min/1.73 m2. However, higher relapse rates were detected in PR3-ANCA positive patients [9]. After induction of remission, maintenance treatment can be continued with low dose corticosteroids, mycophenolate mofetil, azathioprine or rituximab [5].

Separately, chronic recurrent multifocal osteomyelitis (CRMO) is an autoinflammatory disease characterized by sterile bone inflammation. Several monogenic autoinflammatory bone disease conditions exist including Majeed syndrome, deficiency of interleukin-1 receptor antagonist (DIRA), and pyogenic arthritis, pyoderma gangrenosum, and acne syndrome (PAPA) [10]. Its peak age of onset is age 7–12 years, also affecting females more than males [10]. Common clinical manifestations include local bone pain, tenderness, swelling, and warmth [11]. However, pain could be absent, and the patient may present with complications such as vertebral compression fractures [11]. Imaging modalities play a pivotal role in diagnosis. While plain X-rays may show mixed osteolytic and sclerotic lesions, they often appear normal in early stages [12]. Magnetic resonance imaging (MRI) is highly sensitive in early stages and can detect bone edema (on T2) and altered diffusion capacity (on diffusion-weighted imaging [DWI] sequences) before bone erosions and sclerosis become apparent [13]. If the diagnosis remains unclear, bone biopsy can help confirm diagnosis and rule out other conditions such as infections or malignancies. Histological findings are non-specific and tend to vary based on the duration of the lesions. While neutrophils and monocytes are usually prominent in early lesions, lymphocytic and plasma cells infiltrate with varying degrees of sclerosis and fibrosis are usually found later [14]. Treatment includes non-steroidal anti-inflammatory drugs (NSAIDs), corticosteroids, disease-modifying anti-rheumatic drugs such as methotrexate or sulfasalazine, TNF inhibitors, or bisphosphonates [15]. We report a novel case of a child with 4-year duration, NSAID-responsive CRMO who acquired anti-neutrophil cytoplasmic antibody (ANCA)-positive, limited GPA.

## Case presentation

**Fig. 1:**
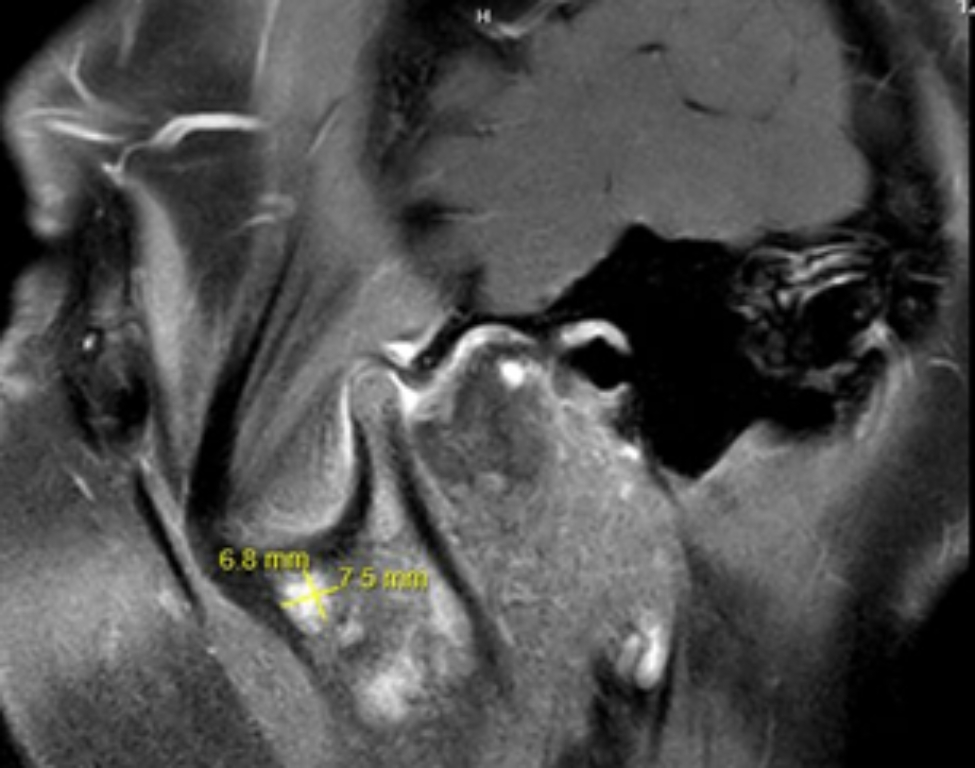
Sagittal oblique fat saturated post open mouth MRI showing T2 hyperintense lesion with enhancement in the subchondral bone extending to the articular cartilage. An additional more ill-defined 6.8 × 7.5 mm lesion is seen anteriorly in the base of the coronoid process

**Fig. 2:**
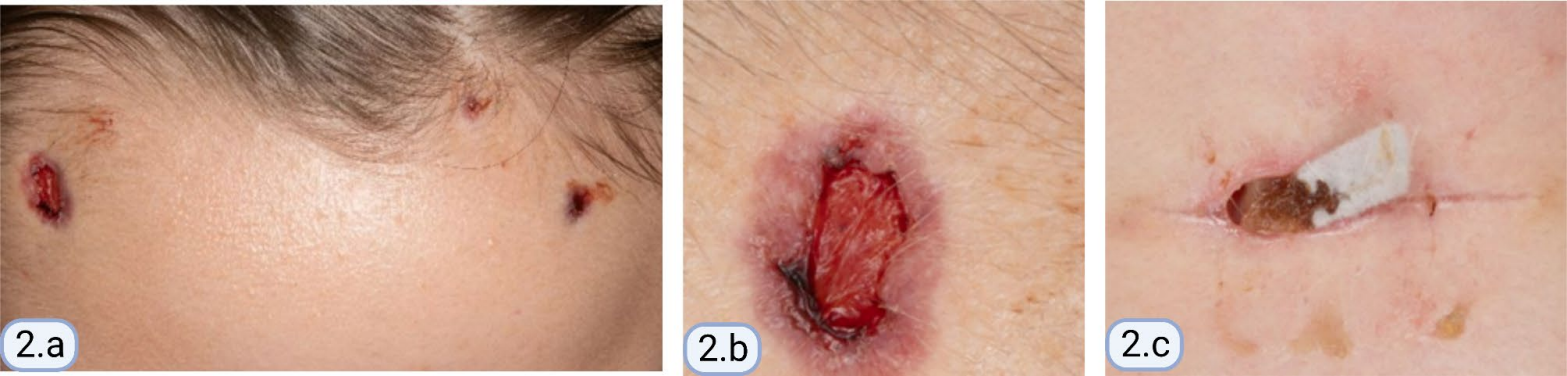
(**a**) Lesions on the forehead, (**b**) close up of a lesion on the forehead, (**c**) lesion on the trunk

**Fig. 3:**
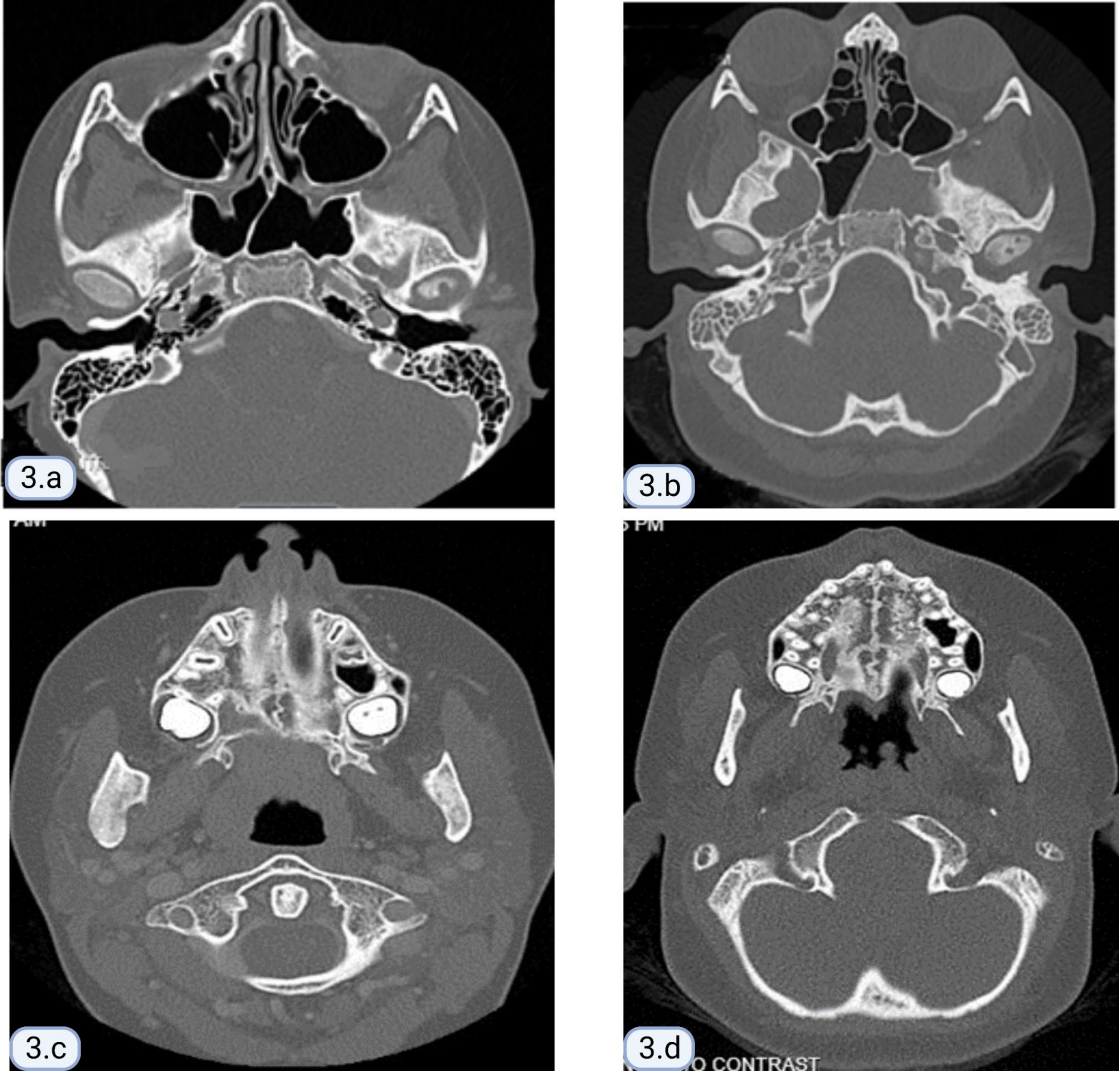
Comparison of CT findings (mastoid air cells-top & mandibular lesion-bottom) between 2018 and 2022. (**a**) CT showing clear mastoid air cells (2018), (**b**) CT showing opacification of the mastoid air cells (2022), (**c**) CT showing expansion of the right mandibular ramus and periosteal reaction (2018), (**d**) CT showing normal mandibular ramus (2022)

**Fig. 4:**
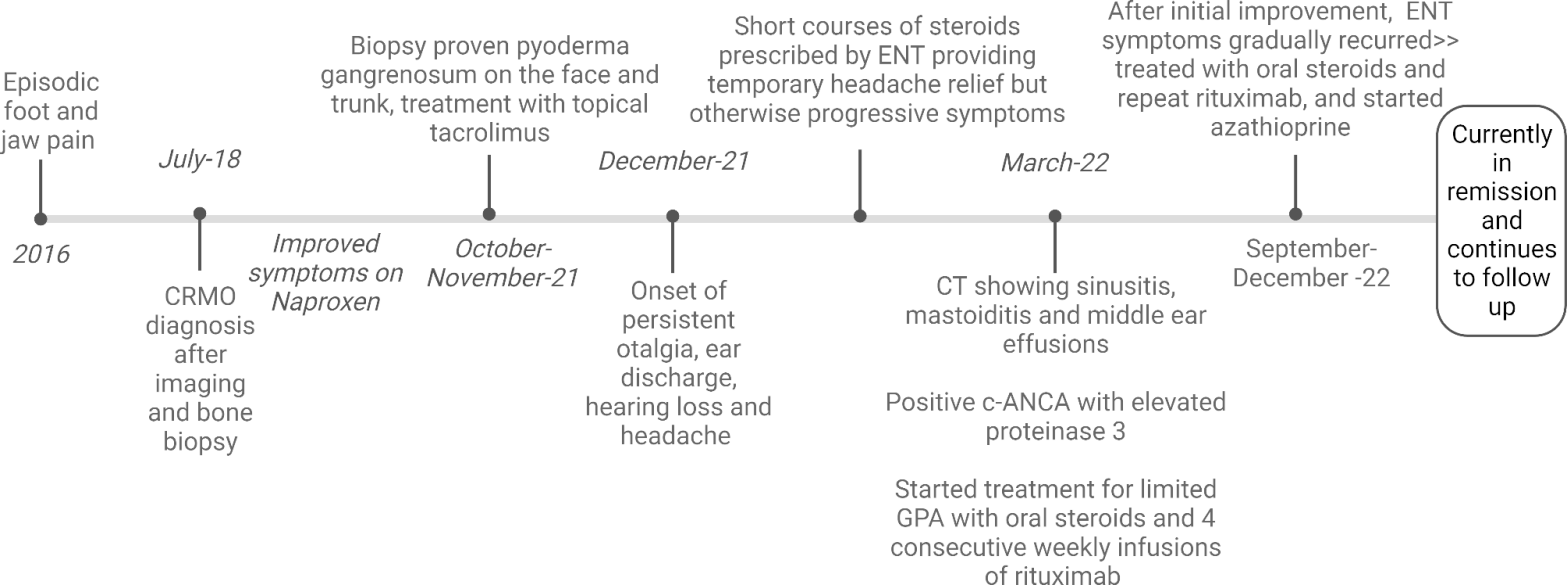
Timeline of the disease course and treatment given

We report a 13-year-old girl, with a family history of mixed connective tissue disease in the mother, who was diagnosed with CRMO in 2018 after presenting with right foot episodic pain and limping. MRI of the temporomandibular joints obtained due to jaw pain, showed abnormal marrow signal within the mandible with several well-defined lesions bilaterally (Fig. 1). Nuclear medicine scan showed low-level FDG uptake in the right elbow and both feet. Bone biopsy of a mandibular lesion revealed sterile chronic osteomyelitis, specifically delicate fibrosis of the bone marrow with scattered clusters of plump plasma cells, positive for CD138. While receiving twice per day naproxen, she experienced an uncomplicated course with effective analgesia. CRMO was under clinical remission with no pain or swelling in the jaws or feet. An elevated but slowly decreasing erythrocyte sedimentation rate (ESR) trend persisted until November 2021 when it increased from 20 to 30 (normal: 0–10 mm/h). One month prior, she developed truncal and forehead lesions that fully responded to topical tacrolimus (Fig. 2). Skin biopsy showed an acute ulcer with dense neutrophilic infiltrate and negative infectious studies, compatible with pyoderma gangrenosum. By December 2021, she developed right otalgia and acute otitis media unresponsive to serial courses of antibiotics, persistent ear discharge, leading to right ear tympanostomy tube placement and left sphenoidotomy. She then developed similar left ear features with gradually progressive hearing loss. Sinusitis ensued without lower respiratory tract symptoms or hematuria. Her ear, nose, and throat (ENT) surgeon prescribed empiric, short courses of prednisone, yielding temporary relief of her marked otalgia and sinus pain. Prednisone withdrawal led to severe otalgia and headache requiring hospitalization. Her head computed tomography (CT) scan indicated left sphenoid sinusitis, bilateral mastoiditis, and middle ear effusions (See Fig. 3 for comparison of CT findings in 2018 and 2022). Chest imaging (radiographs, high resolution CT) and urinalysis were normal. Immunologic studies yielded negative studies for lupus normal complete blood picture, complete metabolic panel, C-reactive protein, and immunoglobulins (A, M and G), with a steadily rising ESR. However, C-ANCA (cytoplasmic-ANCA) was positive with elevated proteinase 3 (PR3) (513 AU/mL) [normal: 0–19 AU/mL]. Sinus biopsy indicated mild chronic inflammation without necrosis, granuloma or vasculitis, a finding potentially confounded by pre-treatment with prednisone. Extensive infectious workup including acid fast bacillus staining, fungal and anerobic cultures was negative. Her clinical features align with limited GPA, acquired during pre-existing CRMO. She received rituximab and oral steroids to induce remission of vasculitis. The patient independently held naproxen in the absence of bone pain. After initial improvement in ear pain, headache, discharge and hearing loss, these symptoms recurred about 6 months after the initial treatment. She was treated with oral steroids and a second round of rituximab and was started on azathioprine. Her disease is currently under adequate control, and she continues to follow up in the pediatric rheumatology clinic. She has not had whole exome sequencing, or any genetic testing performed as it was not considered to be cost effective at that time given the other financial burdens on the family. Of note, there is no family history of consanguinity. (See Fig. 4 for a timeline of the disease course and treatments given).

## Discussion and conclusions

This case report highlights a previously unreported association of limited GPA initially presenting as pyoderma gangrenosum in a patient with underlying CRMO. Reporting these uncommon associations helps guide pediatricians and pediatric rheumatologists in making the appropriate and timely diagnoses in such challenging cases. CRMO usually follows a relapsing and remitting course, although a chronic, unremitting course may occur. This disease predominantly affects children and adolescents; however, it can affect adults [10]. While CRMO typically has spontaneous onset, it can be associated with inherited monogenic autoinflammatory syndromes such as Majeed syndrome, deficiency of interleukin-1 receptor antagonist (DIRA), and pyogenic arthritis, pyoderma gangrenosum, and acne syndrome (PAPA) [10].

CRMO strongly associates with a personal or family history of inflammatory disorders of the skin, joints, or gastrointestinal tract, suggesting a shared pathogenetic pathway. The most prevalent associations are palmar-plantar pustulosis, psoriasis, ankylosing spondylitis, and Crohn’s disease. Familial cases suggest a genetic predisposition [10]. Identified gene mutations in both human and murine models include *LPIN2*, *PSTPIP2*, *IL1RN*, *FBLIM1*, and *MEFV* [16–19].

As a member of the autoinflammatory disorders family, CRMO arises from immune dysregulation leading to activation of the innate immune system. Autoantibodies and, at least initially, autoreactive lymphocytes are absent. While monogenic autoinflammatory syndromes involving CRMO have a known defective pathway, the primary affected pathway in spontaneous onset CRMO remains unknown. Several reports describe an imbalance between pro- and anti-inflammatory cytokines. These include increased expression of apoptosis-associated speck-like protein (ASC), caspase-1 (CASP-1), and interleukin-1 β (IL-1β) messenger RNA in peripheral blood cells from children with active disease versus inactive disease as well as healthy children [20]. An additional report describes significantly lower interleukin-10 (IL-10) production by monocytes in response to T-lymphocyte receptor 4 (TLR 4) activation by lipopolysaccharides [21]. Further, serum levels of interleukin-6 (IL-6), interleukin-12 (IL-12), RANTES, monocyte chemoattractant protein-1 (MCP-1), and soluble interleukin-2 receptor (sIL-2R) are significantly higher in children with active CRMO versus healthy controls, followed by a significant decline in sIL-2R and IL-6 in those achieving complete remission [22].

The cause of GPA is unknown, although like many other polygenic autoimmune diseases, it is likely multifactorial. Genetic factors leading to loss of self-tolerance together with environmental triggers are likely involved [2]. Genetic-association studies of AAV report variants in single nucleotide polymorphisms, with the strongest and most reproducible genetic variants represented in human leukocyte antigen, protein tyrosine phosphatase nonreceptor type 22 (PTPN22), and cytotoxic T-lymphocyte antigen 4 [23]. Studies of environmental triggers suggest AAV can associate with crystalline silica exposure and farming [24], or infectious triggers [25].

As implied by the presence of ANCAs, neutrophils are a key factor in the pathogenesis of GPA. ANCAs in GPA predominantly target PR3 with increased expression of PR3 on the membranes of neutrophils in GPA patients compared to healthy controls [26]. Other mechanisms include activated B-lymphocytes that increase in patients with GPA compared to healthy controls and are higher in patients with active disease versus those in remission [27]. Moreover, response to B-cell depletion therapy suggests a pathogenic role of autoantibodies [27]. However, the presence of granulomas suggests a more complex process. The cell-mediated hypersensitivity model of this disease suggests a predominance of T-helper 1 (Th1) cells [28]. Cytokines may play a role given the inflammatory response to microbial pathogens and environmental exposures [25]. T-helper 17 (Th17) may also have a role in GPA’s pathogenesis [29].

The association of CRMO and GPA is unusual, with a few prior variations reported. A previous case exists of CRMO with systemic GPA involving renal vasculitis and hyperparathyroidism [30]. While observing 14 children with CRMO during a seven-year period, Pelkonen et al. report one of the patients developed a pharyngeal tumor with biopsy features compatible with GPA, accompanied by renal vasculitis [31]. Brogan et al. report a 12-year-old girl with C-ANCA-positive upper respiratory GPA together with multifocal, noninfectious osteitis involving the ribs, acetabulum, and other foci. Bone biopsy indicated extensive fibrosis with a heavy, patchy, mixed inflammatory infiltrate with prominent plasma cells, macrophages, and CD68-positive histiocytes on immunostaining without necrosis. Corticosteroids and rituximab induced remission, followed by maintenance therapy with low dose corticosteroids and azathioprine that led to near-complete resolution and bone remodeling. The authors describe the bony lesions as extracranial skeletal involvement of GPA [32]. This involvement is very rare in GPA, reported as unifocal skeletal lesions and primarily identified in adults [33]. Notably, Brogan’s report describes concomitant GPA with bony lesions, considering bone involvement as part of GPA disease. Our patient received rituximab for limited GPA induction, without requiring corticosteroids. She continues to have an elevated ESR suggestive of an ongoing contribution from her antecedent CRMO. Testing for ANCA did not arise until she manifested features of vasculitis. Lesions attributed by Brogan et al. to bony involvement from GPA do not appear to befit our case, where bone lesions could be attributed to CRMO existing for 4 years prior to her onset of limited GPA. To our knowledge, our case represents the first in which persistently active CRMO preceded the onset of limited GPA by years. It is not completely clear why the CRMO symptoms improved despite discontinuing naproxen. CRMO was already under control on naproxen and its course might have been evolving towards remission by the time she acquired the GPA diagnosis.

The cause of our patient’s complex, co-existing immune dysregulation involving initial autoinflammatory disease followed by autoimmune disease is uncertain. While pathophysiologic mechanisms for both disease entities are different and complex, there is potential for overlapping cytokine pathway dysregulation. Nevertheless, the question remains if the bony lesions could be an early musculoskeletal expression of GPA or are these lesions exclusively consequent to spontaneous-onset CRMO later complicated by limited GPA.

This is the first report of limited GPA initially presenting as pyoderma gangrenosum in a patient with underlying CRMO. The cause of our patient’s complex, co-existing immune dysregulation involving initial autoinflammatory disease followed by autoimmune disease is equally uncertain.

### Electronic supplementary material

Below is the link to the electronic supplementary material.

Supplementary Material 1

## Data Availability

Available.

## List of abbreviations

GPA: granulomatosis with polyangiitis
CRMO: chronic recurrent multifocal osteomyelitis
NSAIDS: non-steroidal anti-inflammatory drugs
ANCA: anti-neutrophil cytoplasmic antibody
AAV: ANCA associated vasculitis
ESR: erythrocyte sedimentation rate
ENT surgeon: ear, nose, and throat (ENT)
CT: computed tomography
C-ANCA: cytoplasmic ANCA
DIRA: deficiency of interleukin-1 receptor antagonist
PAPA: pyogenic arthritis, pyoderma gangrenosum, and acne syndrome
ASC: apoptosis-associated speck-like protein
CASP-1: caspase-1
IL-1β: interleukin-1β
IL-10: interleukin-10
TLR 4: T-lymphocyte receptor 4
IL-6: interleukin-6
IL-12: interleukin-12
MCP-1: monocyte chemoattractant protein-1
sIL-2R: soluble interleukin-2 receptor
PTPN22: protein tyrosine phosphatase nonreceptor type 22
Th1: T-helper 1
Th17: T-helper 17
