# Supplementary material for: Pediatric-onset limited ANCA-associated vasculitis arising during pre-existing chronic recurrent multifocal osteomyelitis
Source: Pediatr Rheumatol Online J. 2023 Aug 24;21:89. doi: 10.1186/s12969-023-00876-x (PMC10463712; doi:10.1186/s12969-023-00876-x)
Supplement: Supplementary file 1 — Supplementary Material 1 [file 12969_2023_876_MOESM1_ESM.docx]

**Dear Editor**

Enclosed herein is an article entitled “Pediatric-onset limited ANCA-associated vasculitis arising during pre-existing chronic recurrent multifocal osteomyelitis*”* for evaluation and possible publication in your esteemed journal. This article is a case report on a 14-year old patient with chronic recurrent multifocal osteomyelitis (CRMO) that was doing well on non-steroidal anti-inflammatory drugs (NSAIDS) for 6 years and then developed ANCA positive limited GPA presenting with pyoderma gangrenosum and persistent otalgia, ear drainage, followed by hearing loss. We believe this manuscript is appropriate for publication in the journal of “Pediatric Rheumatology” because to our knowledge, this is the first report of limited GPA in a patient with underlying CRMO.

We would like to state that the above-mentioned manuscript is our original work and has not been published elsewhere, nor is it currently under consideration for publication elsewhere. All the co-authors listed below have agreed to have the manuscript submitted for publication.

**With regards,**

**Esraa Eloseily, MD**

**(Corresponding author)**

**Michael Henrickson, MD, MPH**
